# Structure and function of rhizosphere soil microbial communities associated with root rot of *Knoxia roxburghii*

**DOI:** 10.3389/fmicb.2024.1424633

**Published:** 2024-07-18

**Authors:** Chunju Liu, Heng Li, Jiahong Dong, Xiahong He, Lei Zhang, Bin Qiu

**Affiliations:** ^1^School of Chinese Materia Medica, Yunnan University of Chinese Medicine, Kunming, Yunnan, China; ^2^College of Plant Protection, Yunnan Agricultural University, Kunming, Yunnan, China; ^3^R&D Center of Yunnan Yuntianhua Co., Ltd., Kunming, Yunnan, China; ^4^School of Landscape Architecture and Horticulture Science, Southwest Forestry University, Kunming, Yunnan, China

**Keywords:** *Knoxia roxburghii*, root rot, rhizosphere microorganism, physicochemical properties, enzyme activities

## Abstract

The microbial communities in rhizosphere soil play important roles in plant health and crop productivity. However, the microbial community structure of rhizosphere soil still remains unclear. In this study, the composition, diversity and function of the microbial communities in the rhizosphere soil of healthy and diseased plants were compared using Illumina MiSeq high-throughput sequencing. The Sobs (richness) and Shannon (diversity) indices of the soil microbial communities were higher in the rhizospheres of 2- and 3-year-old susceptible plants than in those of the healthy plants. With the increase in planting time, the numbers of fungi tended to decrease, while those of the bacteria tended to increase. Fungal diversity could be used as a biological indicator to measure the health of *Knoxia roxburghii*. The microbial composition and differential analyses revealed that the rhizosphere soil infested with fungi had a higher relative abundance at the phylum level in Ascomycota and Basidiomycota, while the bacteria had a higher relative abundance of Chloroflexi and a lower relative abundance of Actinobacteriota. At the genus level, the rhizosphere soil infested with fungi had relatively more abundant *unclassified_f__Didymellaceae* and *Solicoccozyma* and relatively less abundant *Saitozyma* and *Penicillium*. The bacterial genus *norank_f__Gemmatimonadaceae* was the most abundant, while *Arthrobacter* was less abundant. In addition, the abundance of *Fusarium* in the fungal community varied (*p* = 0.001). It tended to increase in parallel with the planting years. Therefore, it was hypothesized that the change in the community composition of *Fusarium* may be the primary reason for the occurrence of root rot in *K. roxburghii*, and the change in the abundance of *Fusarium* OTU1450 may be an indication of the occurrence of root rot in this species. The community function and prediction analyses showed that the pathogenic fungi increased with the increase in planting years. In general, soil fungi can be roughly divided into three types, including pathotrophs, symbiotrophs, and saprotrophs. An analysis of the differences in the prediction of different rhizosphere functions showed that D and L were significantly different in the COG enrichment pathway of the *K. roxburghii* rhizosphere bacteria (*p* < 0.05). The soil physical and chemical properties, including the pH, AK, total potassium (TK), and catalase (S_CAT), had the most significant effect on the soil fungal community, and most of the soil physical and chemical properties significantly correlated with the bacterial community. This study demonstrated that the occurrence of root rot had an important effect on the diversity, structure and composition of microbial communities. In addition, the results will provide a theoretical basis to prevent and control root rot in *K. roxburghii*.

## Introduction

*Knoxia roxburghii* (Sprengel) M. A. Rau, synonym *Knoxia valerianoides* Thorel et Pitard, is a first-grade protected traditional Chinese medicine (TCM) that grows wild in China. The root is used to treat diarrhea, eliminate swellings and disperse knots among others (Liu et al., 2023b). Its radix has diuretic, anti-inflammatory, and antitumor effects (Chen et al., 2022). This plant is primarily distributed in southern China and Southeast Asia (Zhang et al., 2023). The major organic compounds in the extracts of *K. roxburghii* are anthraquinones and triterpenoids. Many anthraquinones have displayed anti-tumor, antibiotic and insecticidal activities (Dikkala et al., 2023). Anthraquinones are also an important source of anti-tumor drugs (Chen et al., 2022). With the increasing incidence of cancer and the modernization of TCM, the demand for *K. roxburghii* root materials is increasing. *K. roxburghii* is susceptible to various plant diseases, and root rot is the most serious. This disease substantially limits its production.

Among plant diseases, root rot is a soilborne disease. Its incidence is closely related to plant health, water, nutrition, soil physical and chemical factors and the microbial community structure of the rhizosphere. It has been reported that many medicinal plants are vulnerable to root rot (Latha et al., 2011; Gordo et al., 2012). Chinese ginseng (*Panax notoginseng*) is particularly susceptible to root rot, with an annual incidence that ranges from 5–20% and can cause severe losses as high 70% (Wu et al., 2015). Similarly, in Canada 20–30% of the annual ginseng crops are lost to root rot (Walsh et al., 2021). Furthermore, the incidence of root rot on *K. roxburghii* is between 10 and 15% (Liu et al., 2023a).

The rhizosphere is considered to be the area that has the greatest impact on plant nutrition and growth. It is defined as a narrow area of soil around the root and is directly affected by microorganisms and root exudates (Mendes et al., 2013; Lakshmanan et al., 2014). The whole system of the interactions between the plant root and rhizosphere microbiome constitutes the plant-root microbiome (Philippot et al., 2013). Rhizosphere microorganisms (Mendes et al., 2013) have formed mutual relations, such as symbiosis, parasitism, mutualism, and antagonism, during the long-term process of evolution (Chaudhary et al., 2024). Some agronomic practices can affect plant-microbial interactions, including continuous cropping (Tan et al., 2017a; Xu et al., 2020; Wang et al., 2022), crop rotation (Lin et al., 2009), intercropping (Noushahi et al., 2021) and irrigation and fertilization (Liu et al., 2015; Xie et al., 2022), as well as the application of pesticide-fertilizer combinations (Huang et al., 2021).

Studies have shown that most of the pathogens that cause root rot are fungi, including *Fusarium oxysporum*, *F. solani*, *Penicillium subrubescens*, and *Rhizoctonia solani* (Latiffah et al., 2010; Scherm et al., 2013; Chittem et al., 2015; Liu et al., 2023a,b), with *Fusarium* as the predominant pathogen. Soil microbial diversity refers to the species and interspecific differences of microbial communities, which reveal the richness of microbial life in the soil and show the quality and potential of the soil (Zhou et al., 2012). Rhizosphere and root-associated microbial communities are closely related to soilborne diseases and plant health. Under the limited consumption of rhizosphere microbial nutrients, only a few rhizosphere microorganisms do not occupy the niche, which provides an opportunity for the invasion of potential soilborne pathogens (Mallon et al., 2015; Wei et al., 2015). Studies have compared the soil of the rhizospheres of healthy and diseased *P. notoginseng*, and the results showed that there was a decrease in the microbial community in the rhizosphere soil and in the alpha-diversity of the roots of diseased plants (Wu et al., 2015). There was less fungal diversity in the rhizosphere soil of diseased *P. notoginseng* than in healthy plants (Tan et al., 2017b). Compared to the healthy rhizosphere, the types of soilborne pathogens, such as *Alternaria*, *Botrytis cinerea*, *Cladosporium*, *Verticillium*, and *Fusarium*, in the rhizosphere soil of susceptible American ginseng (*Panax quinquefolius*) increased, while the diversity of potential beneficial microorganisms, such as *Bacillus* and *Chaetomium*, decreased (Ji et al., 2021). Similarly, the bacterial diversity in the rhizosphere soil of Chinese goldthread (*Coptis chinensis*) root rot was significantly reduced, which may be the primary cause of root rot (Song et al., 2017). There was less diversity in the bacteria found in the rhizosphere of healthy Lanzhou lily (*Lilium davidii* var. unicolor) than that found in susceptible plants, while the fungi were more diverse (Shang et al., 2016). However, when plants are attacked by harmful pathogens, they also take corresponding measures to defend themselves. For example, plants can recruit protective microorganisms and enhance microbial activity to inhibit the growth of rhizosphere pathogens (Berendsen et al., 2012, 2018). Diversified microbial communities can provide a stable and balanced rhizosphere ecosystem. When plants are infected by pathogens, they can improve their resistance to disease by inhibiting these microorganisms (Trivedi et al., 2012). Therefore, the problem of the reduction in the yield of *K. roxburghii* could be improved by identifying the pathogens related to root rot.

However, the characteristics of the rhizosphere microbial community of *K. roxburghii* plants with root rot have not yet been reported. In this study, high-throughput sequencing was used to sequence the microorganisms in the rhizosphere soil of healthy *K. roxburghii* plants that had contracted root rot. The goal was to analyze the composition and variation of microbial communities in the rhizosphere soil of *K. roxburghii* that had succumbed to root rot and healthy plants and quantitatively analyze the physical and chemical properties and enzyme activities of the soil. This research should help to understand the pathogenesis of root rot in *K. roxburghii* and provide a theoretical basis to prevent and treat root rot in this crop.

## Materials and methods

### Sample collection

The field experiment was performed in Xiangyun County, Dali Bai Autonomous Prefecture of Yunnan Province (25°25′ N, 100°40′ E), a prominent region for the cultivation of *K. roxburghii* in China. The occurrence of root rot of *K. roxburghii* was observed in August 2021, with an incidence of approximately 15% (Liu et al., 2023a). The rhizosphere soil of 2- and 3-year-old *K. roxburghii* plants was collected by a diagonal sampling method, and soil that had not been planted with *K. roxburghii* was used as the control. In each plot, the rhizosphere soil of five healthy *K. roxburghii* plants of those with root rot was uniformly selected and mixed, and the soil without *K. roxburghii* was sampled and mixed according to the five-point sampling method. After the soil samples were collected, they were quickly stored on dry ice after the soil had been screened through a 4-mm sieve. During the sampling process, the plants were uprooted, and the excess soil was shaken off. The soil left on the root surface was considered to be the rhizosphere soil (Tan et al., 2017b).

The soil collection and classification were as follows: 2-year-old healthy *K. roxburghii* rhizosphere soil (S_H2), 3-year-old healthy *K. roxburghii* rhizosphere soil (S_H3); the rhizosphere soil of 2-year-old *K. roxburghii* with root rot (S_D2) and the rhizosphere soil of 3-year-old *K. roxburghii* with root rot (S_D3) were studied. Unplanted soil was used as the blank control (S_CK) of this experiment, and there were six replicates of each sample.

### Soil physicochemical properties and enzyme activities

The soil physical and chemical properties that were measured primarily included the soil pH, organic matter (OM), total nitrogen (TN), total phosphorus (TP), total potassium (TK), hydrolytic nitrogen (HN), available phosphorus (AP), and available potassium (AK) (Liu et al., 2024b). The soil enzymes were primarily catalase (S_CAT), urease (S_UE), sucrase (S_SC), and cellulase (S_CL) (Abay et al., 2022). The eight routine tests of the soil are the basic indices of the physical and chemical properties of the soil. These parameters can reflect the basic status of the soil.

### DNA extraction, PCR amplification, and sequencing

The total microbial genomic DNA was extracted from the samples of rhizosphere soil and blank control soil using a PowerSoil Pro DNA Kit (Qiagen, Hilden, Germany) according to the manufacturer’s instructions. The quality and concentration of DNA were assessed by 1% agarose gel electrophoresis and a NanoDrop® ND-2000 spectrophotometer (Thermo Fisher Scientific, Waltham, MA, United States) and stored at −80°C for further analysis. The hypervariable region of the fungal ITS gene was amplified using the primer pairs ITS1-F (5′-CTTGGTCATTTAGAGGAAGTAA-3′) and ITS2-R (5′-GCTGCGTTCTTCATCGATGC-3′) using an ABI GeneAmp® 9700 PCR thermocycler (Applied Biosystems, Waltham, MA, United States) (Adams et al., 2013). The bacterial amplification region was the V3-V4 region using the primers 338F (5′-ACTCCTACGGGAGGCAGCAG-3′) and 806R (5′-GGACTACHVGGGTWTC TAAT-3′) (Liu et al., 2016). The PCR reaction mixture consisted of 5 × Fast Pfu buffer (4 μL), 2.5 mM dNTPs (2 μL), 5 μM each primer (0.8 μL), Fast Pfu polymerase (0.4 μL), and template DNA (10 ng). ddH_2_O was added to bring the final volume to 20 μL. The PCR amplification procedure involved initial denaturation at 95°C for 3 min, followed by 27 cycles of denaturing at 95°C for 30 s, annealing at 55°C for 30 s, and extension at 72°C for 45 s, with a final single extension at 72°C for 10 min, and subsequent cooling at 4°C. All the samples were amplified in triplicate, and the resulting amplicons were extracted from a 2% agarose gel and purified using an AxyPrep DNA Gel Extraction Kit (Axygen Biosciences, Union City, CA, United States) according to the manufacturer’s instructions. The extracted amplicons were quantified using a Quantus™ fluorescence meter (Promega, Madison, WI, United States). The sequencing library was prepared by Illumina (San Diego, CA, United States) according to the manufacturer’s instructions. The prepared library was closely examined to assess its quality and integrity. The DNA was sequenced by Shanghai Majorbio Bio-Pharm Technology Co., Ltd. (Shanghai, China) using an Illumina MiSeq PE300 platform.

### Data processing and statistical analysis

FASTP v. 0.19.6 was used to perform quality control on the original sequences to remove low-quality reads, and FLASH v. 1.2.11 was used for splicing to obtain longer sequences (Magoč and Salzberg, 2011; Chen et al., 2018). UPARSE v. 11 was used for operational taxonomic unit (OTU) clustering after quality control splicing, and the chimeras were removed according to 97% similarity (Edgar, 2013). To minimize the impact of sequencing depth on the subsequent analysis of alpha-diversity and beta-diversity, the number of all the sample sequences was flattened to 20,000. After flattening, the average sequence coverage of each sample was still more than 97%. RDP classifier was used to compare the bacterial Silva 16S rRNA gene database (v. 138) and the fungal UNITE database (v. 8.0) to taxonomically annotate the OTUs. The confidence threshold was 70%, and the community composition of each sample was counted at different levels of species classification. Mothur v. 1.30.2 was used to calculate the alpha-diversity and the Chao and Shannon indices among others (Schloss et al., 2009). Multiple comparisons for the Least Significant Difference (LSD) in a one-way ANOVA (SPSS 16.0) were used to analyze the differences in alpha-diversity between the groups. A non-metric multidimensional scaling (NMDS) analysis based on the Bray-Curtis distance algorithm was used to test the similarity of microbial community structure between the samples, and an analysis of similarity (ANOSIM) non-parametric test was used to analyze whether there was a significant difference in the microbial community structure between the sample groups. A Kruskal-Wallis test was used to test the differences between the groups (Segata et al., 2011). A redundancy analysis (RDA) was used to determine the effects of soil physical and chemical indicators and enzyme activities on the soil microbial community structure. Based on a Spearman correlation of |*r*| > 0.6, *p* < 0.05, the species were selected for a correlation network analysis (Barberán et al., 2012). FUNGuild v.1.0 was used to functionally annotate the fungal sequences, and PICRUSt v.1.1.0 was used for the Cluster of Orthologous Groups (COG) functional annotation of the bacterial OTUs.

## Results

### Composition and alpha-diversity of the rhizosphere microorganisms

**Figure 1:**
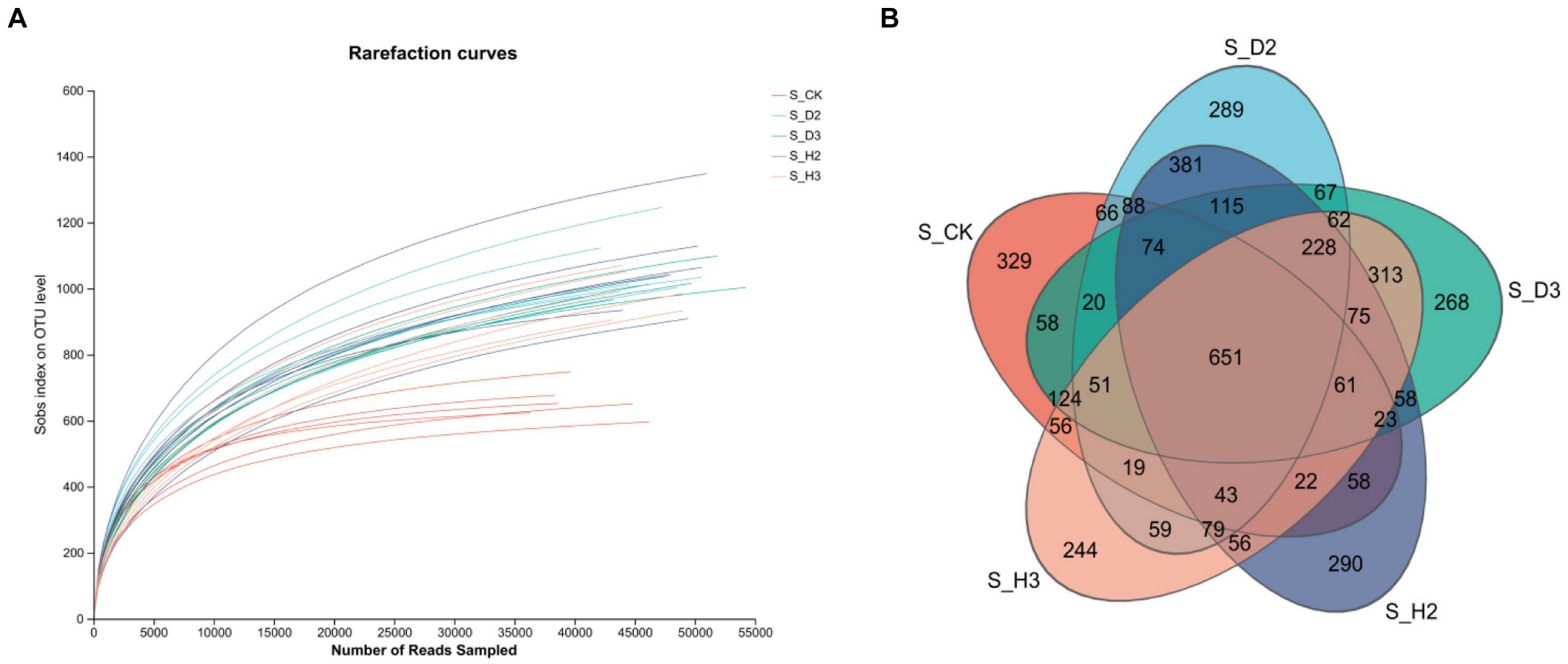
Soil fungal rarefaction curves **(A)** and Venn diagram **(B)**.

The fungal ITS regions of 30 soil samples were sequenced, and 1,419,555 optimized quality control sequences were obtained (Supplementary Table S1). The sequences were 239 bp long on average, and the average sequence after leveling was 33,872 with 97% similarity. All the quality control spliced sequences were subjected to OTU clustering, and there were 29,165 OTUs in total. Among them, there were 651 OTUs in the healthy and root rot rhizosphere compared to the non-planted soil; 381 OTUs in S_H2 and S_D2, and 313 OTUs in S_H3 and S_D3. There were more OTUs in the rhizosphere of healthy plants than that the susceptible ones (Figure 1B).

**Figure 2:**
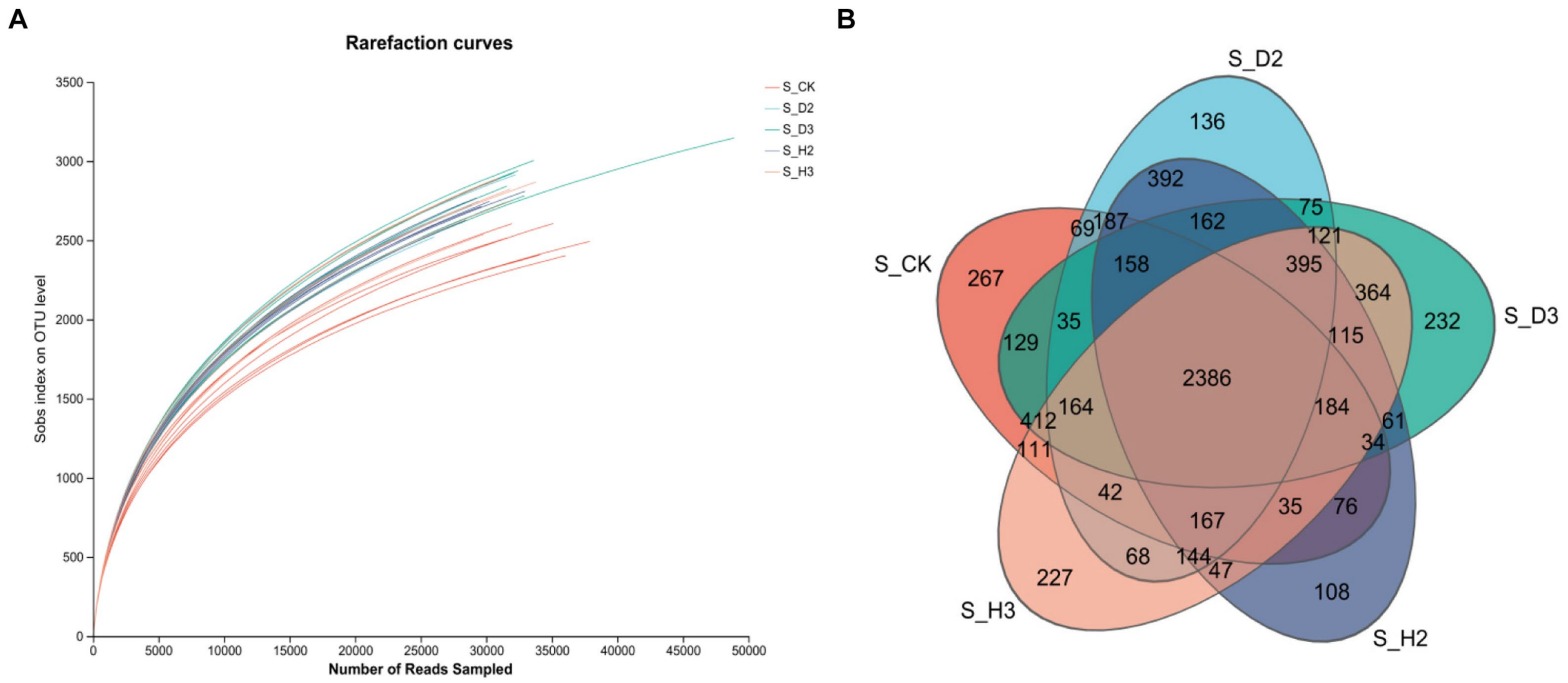
Soil bacterial rarefaction curves **(A)** and Venn diagram **(B)**.

The bacterial 16S V3-V4 regions of 30 soil samples were sequenced. A total of 1,476,629 optimized quality control sequences were obtained by sequencing that were 416 bp long on average (Supplementary Table S2). The average sequence was 26,027, and there were 76,597 OTUs in total. Among them, there were 2,386 OTUs in the healthy and root rot soil compared to the unplanted soil; 392 OTUs in S_H2 and S_D2, and 364 OTUs in S_H3 and S_D3 (Figure 2B).

The dilution curves of each soil sample and the control (Figures 1A, 2A) showed that the number of species of fungi and bacteria increased with the increase in the amount of sequencing data randomly selected, and the curve first increased and then tended to flatten. This indicated that the sequencing amount of the sample can reflect the microbial community structure in the real environment. The dilution curves of the fungi in each treatment were relatively dispersed, which indicated that the fungi among the treatments were highly diverse. The dilution curves between the bacterial treatments were relatively concentrated, which indicated that there was little diversity in the bacteria between the treatments. The curve between the control and each sample sample had some degree of dispersion whether it contained fungi or bacteria. This indicated that there was a substantial difference in the microbial diversity of the control and each soil sample.

The alpha-diversity was estimated by calculating the richness indices, such as Sobs, Chao, Ace, Simpson, and others (Supplementary Table S3). The Good’s coverage of the fungi and bacteria exceeded 97%. Therefore, the rate of detection of the rhizosphere microorganisms of *K. roxburghii* was close to saturation, and the sequencing amount could cover most of the species in the soil. The Sobs (richness) and Shannon (diversity) indices of the soil microbial communities were higher in the rhizospheres of the 2- and 3-year-old susceptible plants than in those of the healthy plants, and the richness indices (Sobs, Ace, and Chao) were significantly higher than those of the unplanted soil (S_CK). With the increase in planting time, the number of fungi tended to decrease, while that of the bacteria tended to increase.

### Structure of the microbial communities

**Figure 3:**
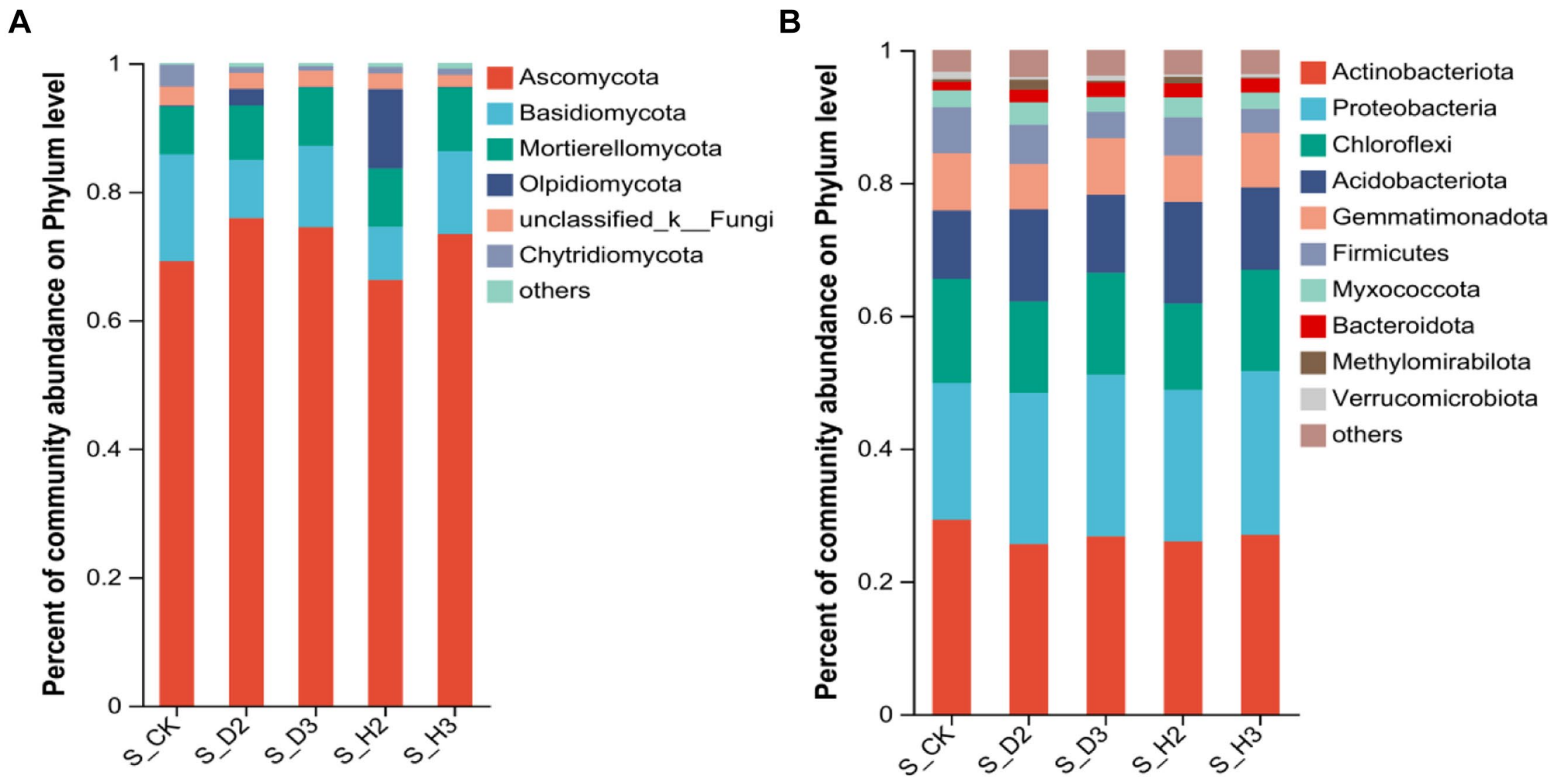
Soil fungal **(A)** and bacterial **(B)** phylum level.

At the phylum level, the distribution of the top 10 species is shown in Figure 3. The five phyla with higher abundances of fungal community were Ascomycota, Basidiomycota, Mortierellomycota, Olpidiomycota, and Chytridiomycota. Among them, the relative abundances of Ascomycota and Basidiomycota in the susceptible rhizosphere soil fungi were higher than those in the healthy rhizosphere (Figure 3A). The 10 phyla of bacteria that were highly abundant included Actinobacteriota, Proteobacteria, Chloroflexi, Acidobacteriota, Gemmatimonadota, Firmicutes, Myxococcota, Bacteroidota, Methylomirabilota, and Verrucomicrobiota (Figure 3B). Among them, Chloroflexi in the susceptible rhizosphere was relatively more abundant, while Actinobacteriota was less abundant.

**Figure 4:**
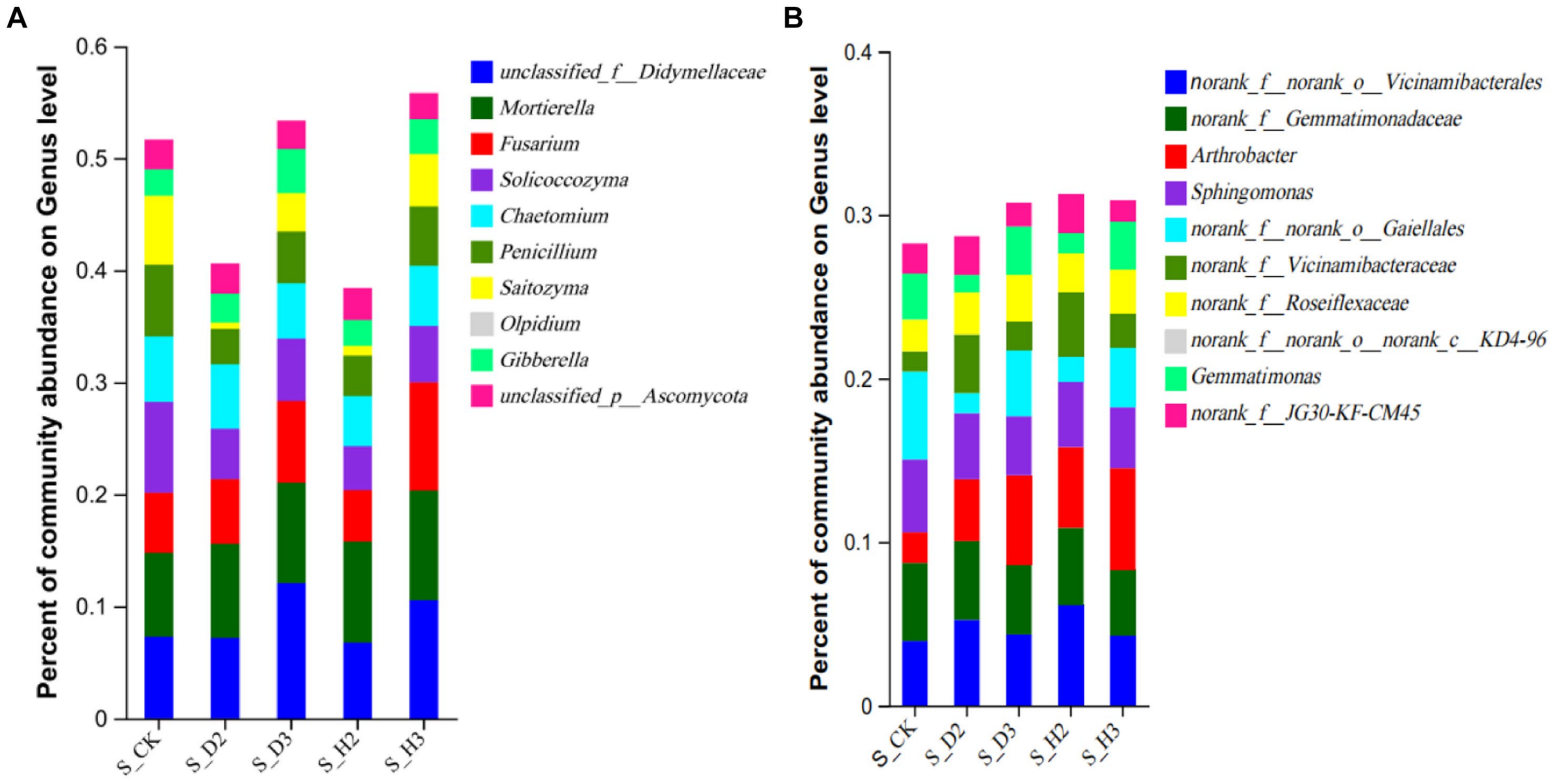
Soil fungal **(A)** and bacterial **(B)** genus level composition.

At the genus level, the distribution of the top 10 species abundances is shown in Figure 4. In all the rhizosphere soil samples, the top 10 genera of fungal community abundance were *unclassified_f__Didymellaceae*, *Fusarium*, *Mortierella*, *Saitozyma*, *Olpidium*, *Solicoccozyma*, *Chaetomium*, and *Penicillium*. Among them, the *Fusarium* in S_D2 was more abundant than that of S_H2, while S_D3 was the opposite. *Fusarium* showed an increasing trend as the number of planting years increased, and the blank control was between 2 and 3 years. Compared to the healthy rhizosphere, the *unclassified_f__Didymellaceae* and *Solicoccozyma* in the susceptible rhizosphere soil fungi were more abundant, while *Saitozyma* and *Penicillium* were less abundant (Figure 4A). The bacterial genera that were more abundant included *Arthrobacter*, *Sphingomonas*, *Gaiellales*, and *Gemmatimonas*. The others were annotated to the corresponding databases and families but not to specific genera, including *Vicinamibacterales*, *Gemmatimonadaceae*, *Vicinamibacteraceae*, *Roseiflexaceae*, and *KD4-96*. Among them, the bacteria *norank_f__Gemmatimonadaceae* were relatively more abundant in the susceptible rhizosphere, while *Arthrobacter* was less abundant (Figure 4B).

**Figure 5:**
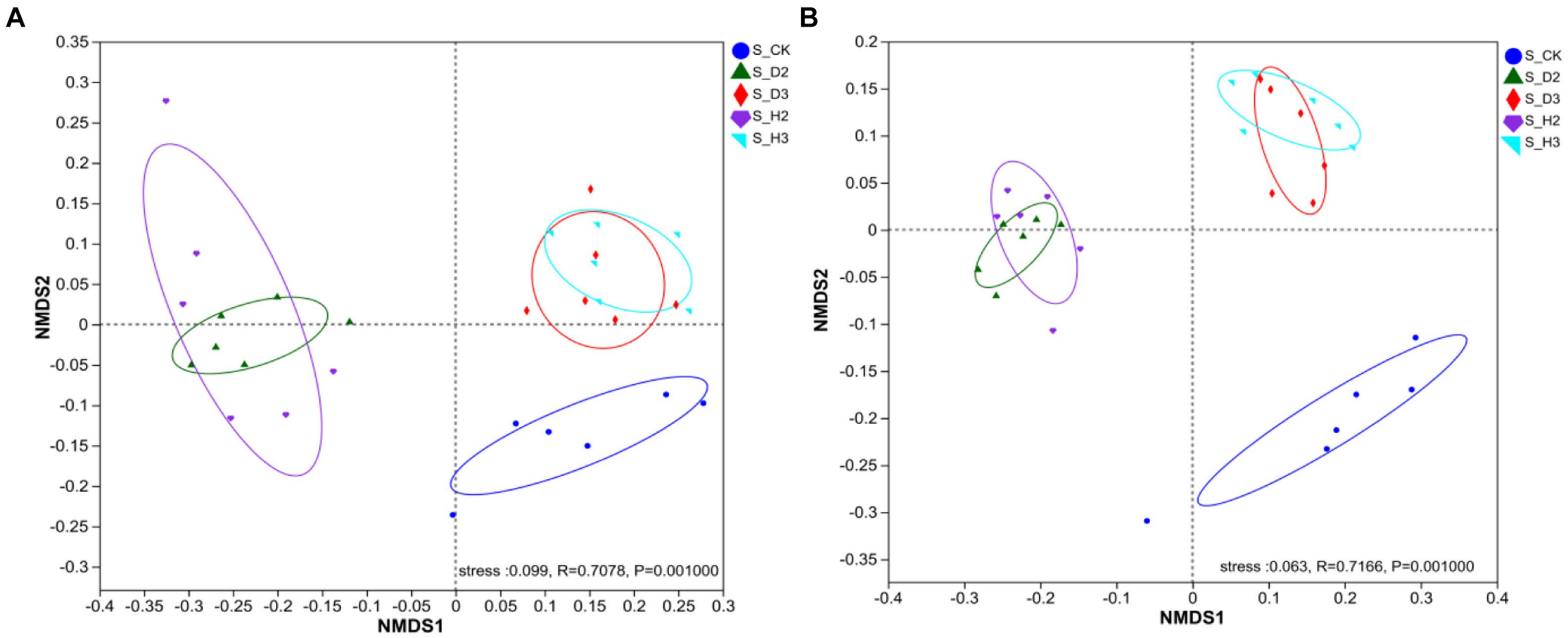
NMDS analysis of soil fungi **(A)** and bacteria **(B)**.

To intuitively represent the differences between different treatments, the NMDS analysis of the soil microorganisms was performed based on the Bray–Curtis dissimilarity. There were significant differences in the fungal community composition among different types of rhizospheres (*R* = 0.7078, *p* = 0.001), which indicated that the fungal community composition was significantly affected by infection of the roots of *K. roxburghii* (Figure 5A). Different bacterial community structures and fungal changes were the same (*R* = 0.7166, *p* = 0.001), and stress = 0.063 (Figure 5B). The microorganisms in the healthy rhizosphere and those that contained pathogens did not overlap and were separated, thus, indicating that the rhizosphere microbial community composition of the susceptible and healthy plants differed. Simultaneously, the rhizosphere microorganisms of the different planting years and unplanted soil were divided into different quadrants. This indicated that the planting years were also factors that caused the differences in the microbial community of *K. roxburghii*.

**Figure 6:**
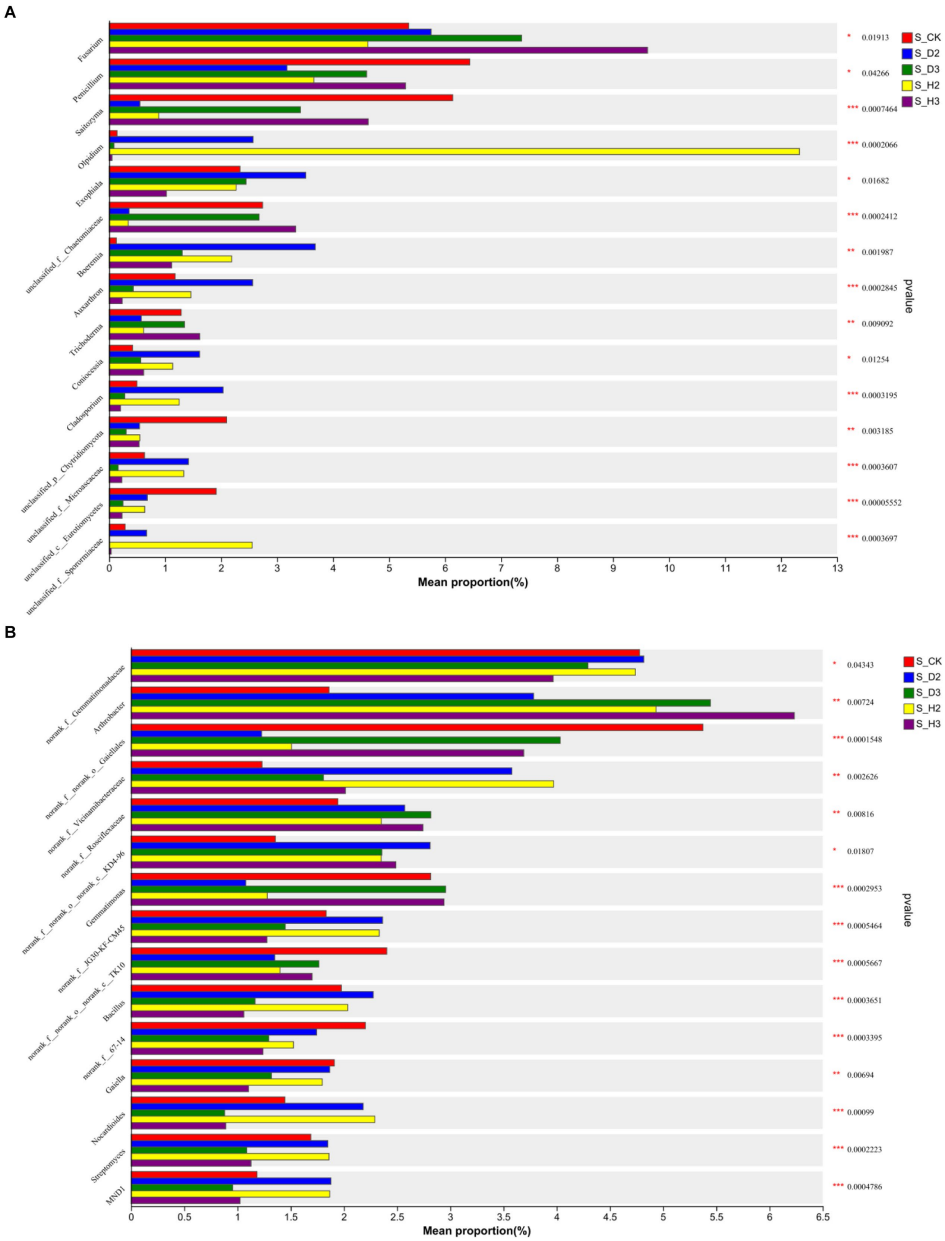
Genus with significant difference between fungi **(A)** and bacteria **(B)**.

Based on the community abundance in each sample, a Kruskal-Wallis test was used to detect the species with different levels of abundance in the 2-year-old, 3-year-old and control soil microbial communities, and a hypothesis test was performed to evaluate the significance of the observed differences. There were significant differences in the four genera of *Fusarium*, *Penicillium*, *Exophiala*, and *Coniocessia* (*p* < 0.05), and there was a substantial change in the abundance of *Fusarium* (Figure 6A). In bacteria, there were significant differences between the *Gemmatimonadaceae* and *KD4-96* communities (*p* < 0.05). There were significant differences among *Arthrobacter*, *Gemmatimonas*, *Bacillus*, *Gaiella*, *Nocardioides*, *Streptomyces*, and eight unannotated genera (*p* < 0.001) (Figure 6B).

**Figure 7:**
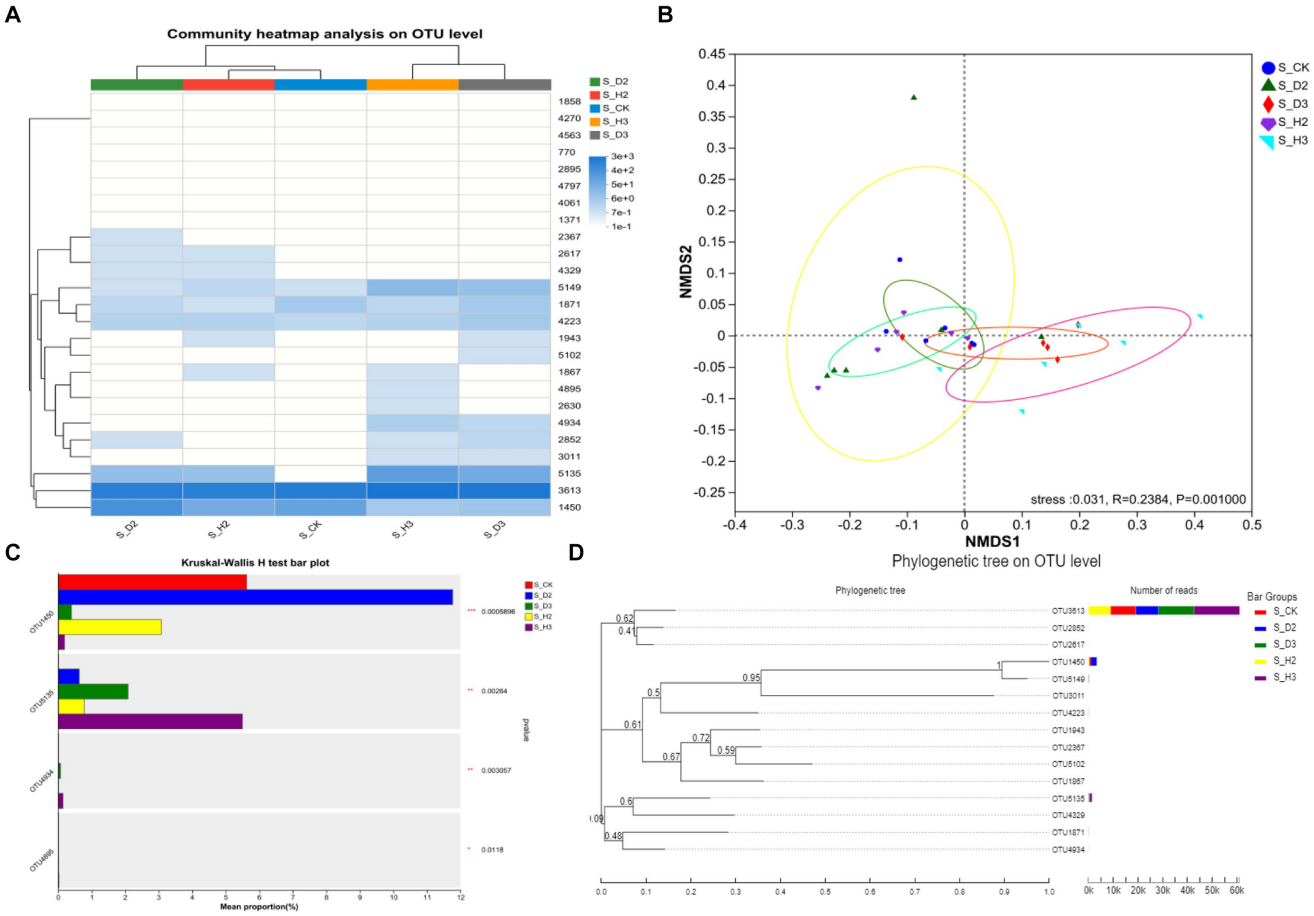
Cluster analysis and difference analysis of the *Fusarium* community in the soil. **(A)** *Fusarium* composition analysis. **(B)** Cluster analysis. **(C)** *Fusarium* community difference analysis. **(D)** Phylogenetic tree.

The structure and difference of *Fusarium* in the rhizosphere fungal community of healthy and infected *K. roxburghii* were analyzed. The primary members of the rhizosphere community of *K. roxburghii* were found to include OTU1450, OTU3613, OTU5135, OTU4223, OTU1871 and OTU5149, and OTU1450 and OTU3613 accounted for the highest proportion (Figure 7A). The NMDS analysis showed that there were significant differences in the community composition of *Fusarium* among the different types of rhizospheres (*R* = 0.2384, *p* = 0.001), and the stress = 0.031 < 0.05, which indicated that the analysis was highly representative. There were significant differences in the *Fusarium* community between the different types of rhizospheres, which indicated that infection of the roots of *K. roxburghii* could significantly affect the community composition of *Fusarium* in the rhizosphere (Figure 7B). The analysis of the difference of the *Fusarium* community in the different types of rhizospheres revealed a significant difference in *Fusarium* OTU1450 between the rhizosphere of healthy *K. roxburghii*, the rhizosphere of *K. roxburghii* with root rot and S_CK (*p* < 0.001), and S_D2 and S_D3 was significantly higher than that of healthy rhizosphere; OTU5135, OTU4934, and OTU4895 were significantly different (*p* < 0.05) (Figure 7C). The representative sequences of *Fusarium* OTUs in the top 15 of high abundance were selected for a phylogenetic analysis, and the phylogenetic tree was constructed using the neighbor-joining method. The results showed that *Fusarium* OTU1450 and OTU5149 were on the same evolutionary branch, which indicated that the two were closely related. *Fusarium* OTU3613 had the highest proportion of Reads in the rhizosphere of each branch, followed by OTU1450 and OTU5135 (Figure 7D).

### Correlation between soil properties, enzyme activity, and the microbial communities

**Figure 8:**
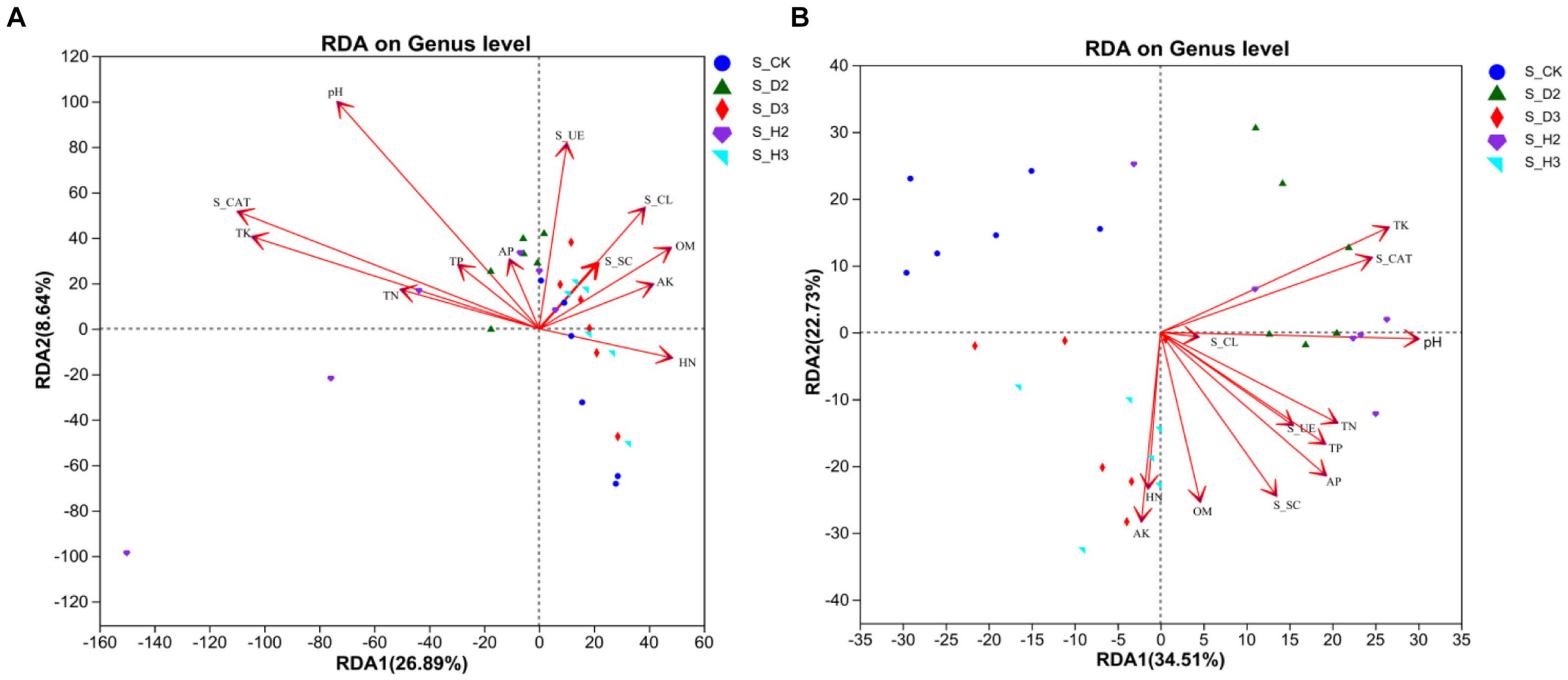
The relationship between soil fungi **(A)** and bacteria **(B)** and environmental variables.

In this investigation, an RDA was used to study the correlation between soil properties, enzyme activity and the microbial community composition of the rhizosphere. The results showed that the response of rhizosphere microbial community composition to soil properties followed a linear model (Axis_lengths <3.5). Therefore, the relationship between the microbial community and soil environmental factors was studied by an RDA (Figure 8). The results of the fungal community showed a correlation between soil environmental factors, such as pH, TP, TK, TN, and S_CL, and there was also a correlation between OM, HN, AK, S_UE, and S_SC. The rhizosphere fungal community of the infected roots positively correlated with S_UE and S_CL, and the healthy rhizosphere positively correlated with OM and HN. In addition, the pH (*p* = 0.001), AK (*p* = 0.001), TK (*p* = 0.001), and S_CAT (*p* = 0.001) significantly correlated with the fungal community structure (Figure 8A). The results of the RDA showed that the rhizosphere bacterial community of the unplanted *K. roxburghii* negatively correlated with the environmental factors, and the rhizosphere bacterial community of 3-year-old *K. roxburghii* positively correlated with the TN and AK. In addition, the rhizosphere of S_D2 positively correlated with the TK, S_CAT, and pH. In addition, there was a significant correlation between the pH, OM, TP, TK, TN, HN, AK, AP, S_UE, S_SC, and S_CAT and the bacterial community structure (*p* < 0.001) (Figure 8B).

**Figure 9:**
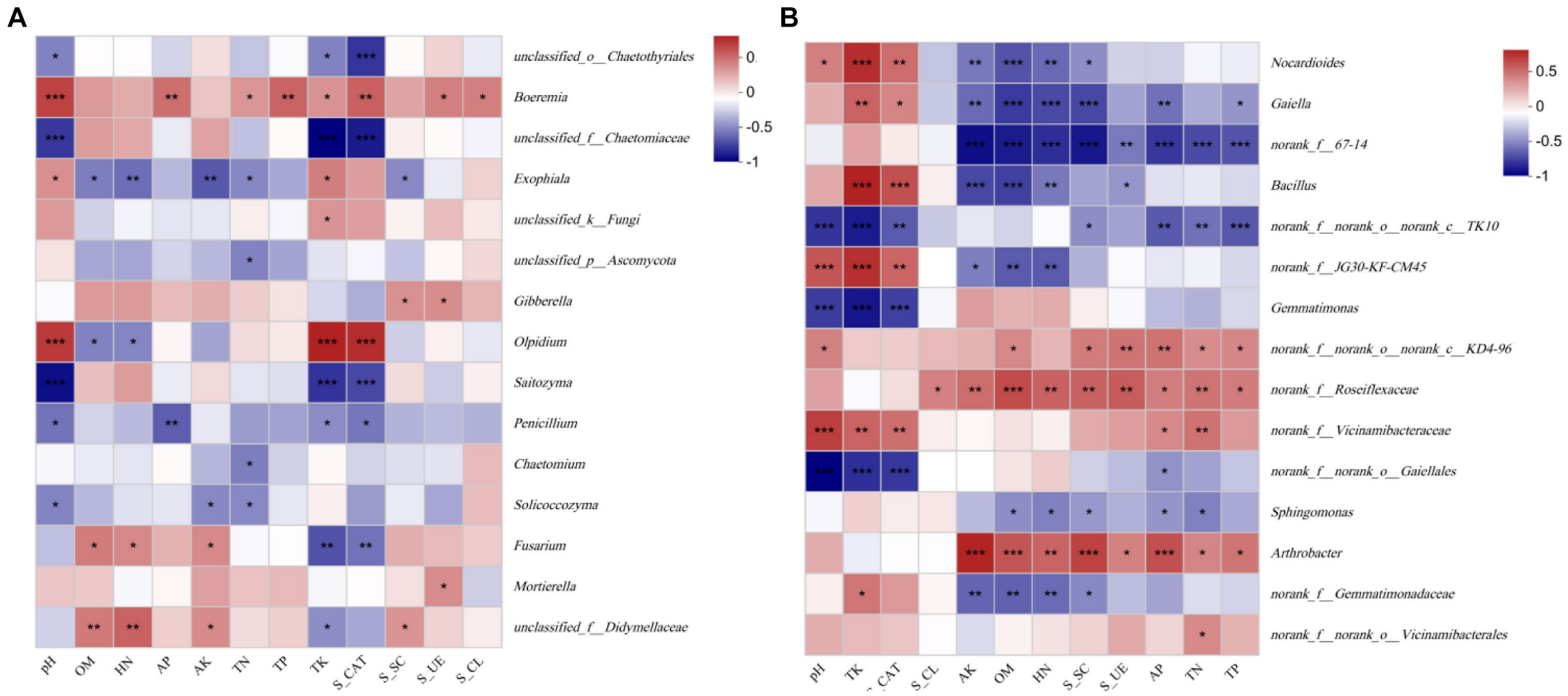
Spearman correlation test of the soil fungi **(A)** and bacteria **(B)**.

The Spearman correlation between the soil physical and chemical properties, enzyme activity and the top 15 species at the genus level was significant as shown in Figure 9. In the fungal community, *Fusarium* sp. positively correlated with the AK (*p* < 0.05), HN (*p* < 0.05) and OM (*p* < 0.01) and negatively correlated with the TK (*p* < 0.01) and S_CAT (*p* < 0.05). *Penicillium* sp. positively correlated with the pH (*p* < 0.05), TK (*p* < 0.05), AP (*p* < 0.01) and S_CAT (*p* < 0.05). *Boeremia* sp. positively correlated with the pH (*p* < 0.001), OM (*p* < 0.05), TP (*p* < 0.01), TN (*p* < 0.05), AP (*p* < 0.01), S_CL (*p* < 0.05), S_UE (*p* < 0.01), and S_CAT (*p* < 0.01) (Figure 9A). A Spearman correlation analysis showed that *Nocardioides* sp., *Gaiella* sp., and *Bacillus* sp. negatively correlated with the AK (*p* < 0.001), HN (*p* < 0.01), OM (*p* < 0.001), and the difference was significant. They positively correlated with the TK (*p* < 0.001) and S_CAT (*p* < 0.001). *Gemmatimonas* sp. negatively (*p* < 0.001) correlated with the pH, AP, and S_CAT, while *Arthrobacter* sp. positively correlated with most of the environmental factors (Figure 9B).

### Prediction of the microbial community function

**Figure 10:**
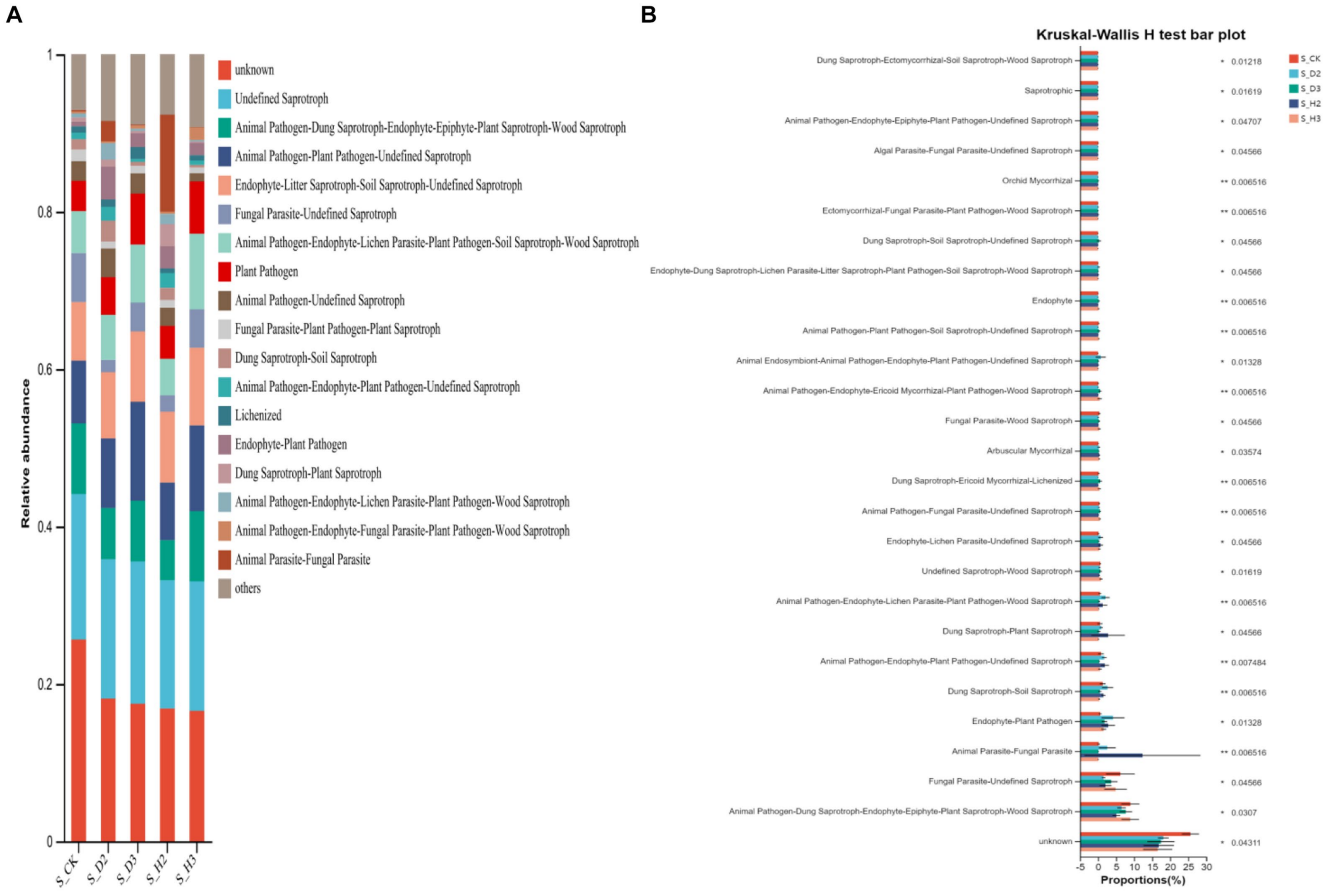
Soil fungi community function prediction **(A)** and difference analysis **(B)**.

The fungal community in rhizosphere soil was classified and analyzed by FUNGuild to obtain the functional classification of fungi in the rhizosphere samples and the abundance information of each functional classification in the different samples (Figure 10A). In general, soil fungi can be roughly divided into three types, including pathotrophs, symbiotrophs, and saprotrophs. The analysis of the differences in the prediction of different rhizosphere functions showed the plant pathogenic bacteria in the rhizosphere of *K. roxburghii* were more abundant than those in the unplanted control. There were more OTUs of plant pathogens in the rhizosphere of S_D2 than in S_H2, and the OTUs of plant pathogens in the rhizosphere of 3-year-old *K. roxburghii* were higher than those in the two-year-old *K. roxburghii*. It was predicted that the pathogenic fungi increased with the increase in planting years. The relative abundances of endophyte-plant pathogen, dung saprotroph-soil saprotroph, dung saprotroph-plant saprotroph, and endophyte in the rhizosphere of susceptible *K. roxburghii* was higher than that in the rhizosphere of healthy *K. roxburghii* (Figure 10B).

**Figure 11:**
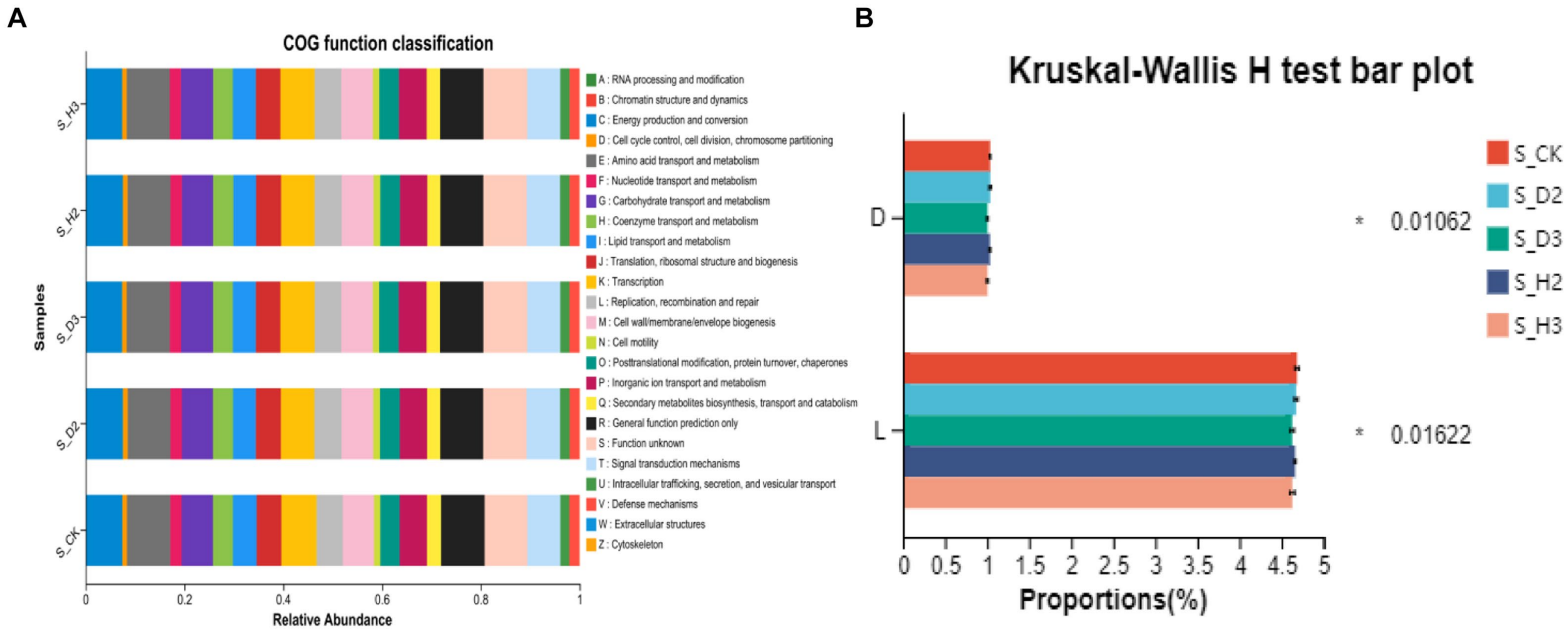
Soil bacterial community function prediction **(A)** and difference analysis **(B)**.

The OTU abundance table was standardized by PICRUSt, and the COG function annotation was performed on the OTUs through the Greengenes id, which corresponded to each OTU to obtain the annotation information of the OTU at the COG function level and the abundance information of each function in different samples. The COG function analysis of the rhizosphere and control of *K. roxburghii* with different planting years showed that there were 24 enriched pathways (Figure 11A). The analysis of the differences in the prediction of different rhizosphere functions showed that D and L were significantly different in the COG enrichment pathway of the *K. roxburghii* rhizosphere bacteria (*p* < 0.05) (Figure 11B). Thus, there were higher populations of bacteria in the susceptible rhizosphere than the healthy rhizosphere, and the soil bacterial community of *K. roxburghii* was not significantly different in the other pathways.

## Discussion

The occurrence of root diseases is related to soil fertility, its structural degradation and the microbial community composition of the soil (Liao et al., 2022). Microbial diversity is an important indicator to measure the soil conditions (Chouhan et al., 2023). *Knoxia roxburghii* is a type of TCM with rhizomes as medicine. Its sustainable production requires an in-depth understanding of the structure and diversity of the rhizosphere microbial communities. In this study, high-throughput sequencing was used to evaluate the changes in the fungal and bacterial communities in the rhizosphere soil of *K. roxburghii* after the plants had developed root rot. The results showed that the occurrence of root rot had a significant effect on the diversity, structure and composition of the fungal and bacterial communities in the rhizosphere soil. The richness index (Sobs) and diversity index (Shannon) of the soil microbial communities in the rhizosphere of S_D2 and S_D3 were higher than those in the healthy rhizosphere, and the richness index (Sobs, Ace and Chao) was significantly higher than that in the unplanted rhizosphere. Therefore, the alpha-diversity of fungi and bacteria in the rhizosphere soil increased after the infection of *K. roxburghii*, and the microbial diversity of the soil without planting *K. roxburghii* was the lowest. The results of this study differ from those of previous studies on the microbial community of root rot in *P. notoginseng* (Wu et al., 2015). Higher soil levels of microbial communities are usually associated with resistance to pathogens. With the increase in planting time, the fungi tended to decrease, while the bacteria tended to increase, which may be the cause of root rot (Tan et al., 2017a,b). Compared to susceptible plants, there was relatively low fungal diversity in the rhizosphere soil of healthy plants. The decrease in the diversity of rhizosphere fungi, particularly the decrease of beneficial groups, may lead to the occurrence of diseases during continuous cropping (Ji et al., 2021). Currently, PICRUSt and FUNGuild are widely used to predict the functional study of microbial communities (Zhang et al., 2016; Schmidt et al., 2019). In the prediction of bacterial function, only two pathways in the 24 pathways of COG function in the rhizosphere of healthy and infected plants differed significantly at a low level. The prediction of fungal function showed that the plant pathogenic bacteria in the rhizosphere of *K. roxburghii* were more abundant than in the unplanted soil, and the OTUs of the plant pathogenic bacteria of 2-year-old plants with root rot were higher than those of the healthy rhizosphere, and the pathogenic fungi increased with the increase in planting years. Therefore, the diversity of fungal communities in the rhizosphere soil of *K. roxburghii* can be used as a biological indicator to measure the health of *K. roxburghii*.

Ascomycota and Basidiomycota are key decomposers in the soil fungal community and are the dominant phyla in all the rhizosphere soils. Simultaneously, their ecological importance was also confirmed in a previous study (Dai et al., 2022). Actinobacteria, Proteobacteria, Chloroflexi, and Acidobacteria were dominant in the rhizosphere of *K. roxburghii*, which was consistent with the results of previous studies on *P. notoginseng* (Tan et al., 2017a,b). At the genus level, with the increase in planting years, *Fusarium* showed an increasing trend. *Fusarium* is an important plant pathogen (Stępień, 2023) and includes such important pathogens as *F. oxysporum* and *F. solani* (Liang et al., 2024; Liu et al., 2024a). Continuous planting resulted in an increase in rhizosphere anthracnose (Jiao et al., 2019). The disturbance of the rhizosphere microbial community and particularly the proliferation of potential pathogens may contribute to the transformation of rhizosphere microenvironment from “healthy” to “diseased” (Wang et al., 2018). Simultaneously, the composition of the *Fusarium* community in infested, healthy and control soils was quite different, and *Fusarium* OTU1450 had a highly significant difference among these soils (*p* < 0.001). The change in the relative abundance of OTU1450 may be a sign of root rot in *K. roxburghii*. This pathogen was found at significantly higher levels in the rhizosphere of infected plants than that of the healthy plants. This indicated that the change in the composition of *Fusarium* in the rhizosphere microbial community of *K. roxburghii* may be the primary reason for the occurrence of root rot in this plant. Therefore, the change of microbial community in the rhizosphere soil, particularly the increase in the abundance of *Fusarium* indicated that there was a potential correlation between the development of root rot and *K. roxburghii* with different planting years.

Microbial diversity, including species composition and interspecific variation within the microbial community, reflects the richness of soil microbial life and is an indicator of soil quality and restoration potential (Liu et al., 2024b). Soil enzymes are important biological indices that reflect the material and energy metabolism and soil quality in soil. S_CAT, S_UE, S_SC and S_CL play an important role in the circulation and transformation of soil nitrogen, carbon and other nutrients and plant protection (Abay et al., 2022). There was less S_UE activity in the rhizosphere of susceptible plants than in that of the healthy plants, which was consistent with the results of previous studies (Wang et al., 2017). In this study, there was a significant correlation between the pH, AK, TK, S_CAT and the fungal community structure. *Fusarium* and *Penicillium* correlated with S_CAT, and it has been previously reported that they are the pathogens that cause root rot in *K. roxburghii* (Liu et al., 2023a,b). Except for S_CL, the bacterial community significantly correlated with most of the environmental factors. The soil pH has a profound influence on the chemical and physical properties of soil and is the primary factor that affects the composition of the rhizosphere soil microbial community (Dhaigude et al., 2024). In this study, the characteristics of rhizosphere microbial community of *K. roxburghii* root rot were identified to provide theoretical support for the prevention and control of this disease. However, the mechanism of root rot merits further study.

## Conclusion

In this study, the microbial community structure of healthy and infested rhizosphere soil and unplanted soil was clarified by high-throughput sequencing. After the infection that caused root rot, there was an increase in the alpha-diversity of the fungi and bacteria in the rhizosphere soil, and the unplanted soil had the lowest amount of microbial diversity. With the increase in planting time, the fungi tended to decrease, while the bacteria tended to increase. Fungal diversity can be used as a biological indicator to measure the health of *K. roxburghii*. The occurrence of root rot of *K. roxburghii* affected the diversity, structure and composition of the fungal and bacterial communities in the soil. The change in the community composition of *Fusarium* may be the primary reason for the occurrence of root rot in *K. roxburghii*, and the change in the abundance of *Fusarium* OTU1450 in the rhizosphere of *K. roxburghii* may be a sign of the occurrence of root rot in this crop. There was a certain correlation between the soil environmental factors and the rhizosphere microbial community of *K. roxburghii*. Future research should focus on the resistance of the microbial community to the root rot of *K. roxburghii* and explore ways to improve the potential for the disease resistance and yield of *K. roxburghii* through the use of targeted microbial communities.

## Data availability statement

The original data have been deposited into the NCBI SRA database with the accession number PRJNA981433 (fungi) and PRJNA991088 (bacteria).

## Author contributions

CL: Writing – original draft, Data curation, Formal analysis, Investigation. HL: Investigation, Writing – review & editing. JD: Project administration, Writing – review & editing. XH: Resources, Writing – review & editing. LZ: Formal analysis, Writing – review & editing. BQ: Funding acquisition, Writing – review & editing.
