# Supplementary material for: Structure and function of rhizosphere soil microbial communities associated with root rot of Knoxia roxburghii
Source: Front Microbiol. 2024 Jul 18;15:1424633. doi: 10.3389/fmicb.2024.1424633 (PMC11291326; doi:10.3389/fmicb.2024.1424633)
Supplement: Supplementary file 1 [file Table_1.DOCX]

Supplementary Table 1 High-throughput sequencing library of soil fungal DNA

| Sample Name | Raw CCS | Clean CCS | Effective sequence | Base_num | Mean_length (bp) | OTU_num |
| --- | --- | --- | --- | --- | --- | --- |
| S_CK_1 | 36712 | 36348 | 33872 | 9106102 | 248 | 629 |
| S_CK_2 | 38764 | 38359 | 33872 | 9475902 | 244 | 678 |
| S_CK_3 | 39024 | 38654 | 33872 | 9596469 | 246 | 656 |
| S_CK_4 | 40541 | 39681 | 33872 | 9814918 | 242 | 755 |
| S_CK_5 | 45806 | 44852 | 33872 | 10816238 | 236 | 670 |
| S_CK_6 | 46765 | 46228 | 33872 | 11183300 | 239 | 605 |
| S_D2_1 | 50117 | 48607 | 33872 | 11939997 | 238 | 1014 |
| S_D2_2 | 52225 | 50578 | 33872 | 12503400 | 239 | 1046 |
| S_D2_3 | 45819 | 44561 | 33872 | 10756379 | 235 | 1029 |
| S_D2_4 | 43965 | 42200 | 33872 | 10609812 | 241 | 1139 |
| S_D2_5 | 49849 | 47313 | 33872 | 11895537 | 239 | 1264 |
| S_D2_6 | 41726 | 40737 | 33872 | 9979202 | 239 | 981 |
| S_D3_1 | 53676 | 51915 | 33872 | 12889424 | 240 | 1110 |
| S_D3_2 | 51449 | 49788 | 33872 | 12475105 | 242 | 1033 |
| S_D3_3 | 49367 | 47597 | 33872 | 11653459 | 236 | 1050 |
| S_D3_4 | 55822 | 54283 | 33872 | 13423416 | 240 | 1012 |
| S_D3_5 | 44892 | 43189 | 33872 | 10697491 | 238 | 966 |
| S_D3_6 | 47405 | 46170 | 33872 | 11196290 | 236 | 1017 |
| S_H2_1 | 52523 | 50617 | 33872 | 12453264 | 237 | 1077 |
| S_H2_2 | 50615 | 49461 | 33872 | 11351608 | 224 | 918 |
| S_H2_3 | 49448 | 47997 | 33872 | 11665562 | 236 | 1062 |
| S_H2_4 | 53469 | 51016 | 33872 | 12741602 | 238 | 1367 |
| S_H2_5 | 51997 | 50299 | 33872 | 12197233 | 235 | 1135 |
| S_H2_6 | 45130 | 44057 | 33872 | 10912643 | 242 | 945 |
| S_H3_1 | 44512 | 43156 | 33872 | 10696732 | 240 | 913 |
| S_H3_2 | 45634 | 43959 | 33872 | 10957211 | 240 | 1078 |
| S_H3_3 | 45740 | 44333 | 33872 | 11174778 | 244 | 1060 |
| S_H3_4 | 45280 | 43818 | 33872 | 10787095 | 238 | 1002 |
| S_H3_5 | 50839 | 49120 | 33872 | 11657163 | 229 | 1011 |
| S_H3_6 | 50444 | 49017 | 33872 | 11782932 | 234 | 943 |
| **Sum** | **1419555** | **1377910** | **1016160** | **338390264** | **239** | **29165** |

Supplementary Table 2 High-throughput sequencing library of soil bacteria DNA

| Sample Name | Raw CCS | Clean CCS | Effective sequence | Base_num | Mean_length (bp) | OTU_num |
| --- | --- | --- | --- | --- | --- | --- |
| S_CK_1 | 49764 | 31600 | 26027 | 20682384 | 416 | 2361 |
| S_CK_2 | 44827 | 31964 | 26027 | 18683888 | 417 | 2437 |
| S_CK_3 | 46081 | 36061 | 26027 | 19222388 | 417 | 2155 |
| S_CK_4 | 45141 | 35106 | 26027 | 18794213 | 416 | 2352 |
| S_CK_5 | 45685 | 37898 | 26027 | 19001934 | 416 | 2189 |
| S_CK_6 | 40257 | 34085 | 26027 | 16715081 | 415 | 2211 |
| S_D2_1 | 51241 | 28929 | 26027 | 21342854 | 417 | 2629 |
| S_D2_2 | 49858 | 29418 | 26027 | 20813825 | 417 | 2614 |
| S_D2_3 | 52603 | 32224 | 26027 | 21941518 | 417 | 2702 |
| S_D2_4 | 52179 | 28899 | 26027 | 21778319 | 417 | 2605 |
| S_D2_5 | 53037 | 27414 | 26027 | 22088088 | 416 | 2622 |
| S_D2_6 | 47096 | 26027 | 26027 | 19662351 | 417 | 2521 |
| S_D3_1 | 50469 | 32108 | 26027 | 20954016 | 415 | 2712 |
| S_D3_2 | 48847 | 33631 | 26027 | 20284076 | 415 | 2746 |
| S_D3_3 | 54634 | 31567 | 26027 | 22724078 | 416 | 2656 |
| S_D3_4 | 74933 | 48904 | 26027 | 31159582 | 416 | 2532 |
| S_D3_5 | 51519 | 32901 | 26027 | 21467553 | 417 | 2559 |
| S_D3_6 | 44635 | 32431 | 26027 | 18543422 | 415 | 2713 |
| S_H2_1 | 49960 | 30247 | 26027 | 20857411 | 417 | 2574 |
| S_H2_2 | 47107 | 29243 | 26027 | 19648146 | 417 | 2654 |
| S_H2_3 | 51939 | 28431 | 26027 | 21631225 | 416 | 2546 |
| S_H2_4 | 49323 | 26225 | 26027 | 20576000 | 417 | 2623 |
| S_H2_5 | 53355 | 29643 | 26027 | 22268123 | 417 | 2580 |
| S_H2_6 | 46876 | 32957 | 26027 | 19563128 | 417 | 2600 |
| S_H3_1 | 43818 | 33802 | 26027 | 18225405 | 416 | 2648 |
| S_H3_2 | 42067 | 30969 | 26027 | 17489488 | 416 | 2726 |
| S_H3_3 | 49283 | 30941 | 26027 | 20492134 | 416 | 2715 |
| S_H3_4 | 49830 | 31897 | 26027 | 20717135 | 416 | 2545 |
| S_H3_5 | 42977 | 31833 | 26027 | 17855340 | 415 | 2648 |
| S_H3_6 | 47288 | 29806 | 26027 | 19639805 | 415 | 2422 |
| **Sum** | **1476629** | **957161** | **780810** | **614822910** | **416** | **76597** |

Supplementary Table 3 The diversity indexes of rhizosphere at Genus level (n=6)

| Source of  sample | Sample | Sobs | Shannon | Ace | Chao | Coverage |
| --- | --- | --- | --- | --- | --- | --- |
| Bacteria | S_D2 | 2615.50±57.88a | 6.53±0.05a | 3681.61±89.22a | 3684.00±101.06b | 0.9682 |
|  | S_D3 | 2653.00±88.56a | 6.45±0.03abc | 3920.03±305.87a | 3852.96±80.00a | 0.9722 |
|  | S_H2 | 2596.17±38.34a | 6.48±0.06ab | 3816.07±309.85a | 3689.82±77.18ab | 0.9736 |
|  | S_H3 | 2617.33±115.43a | 6.43±0.13bc | 3921.75±364.52a | 3730.41±181.27ab | 0.9684 |
|  | S_CK | 2284.17±113.98b | 6.37±0.05c | 3242.30±381.47b | 3119.05±204.57c | 0.9699 |
| Fungi | S_D2 | 330.00±19.43a | 4.01±0.14a | 369.98±28.58a | 369.71±28.26a | 0.9985 |
|  | S_D3 | 325.00±6.48a | 3.75±0.12ab | 366.07±16.00a | 362.08±10.76a | 0.9984 |
|  | S_H2 | 316.17±33.76a | 3.75±0.41ab | 351.46±40.35a | 348.25±40.58a | 0.9986 |
|  | S_H3 | 314.33±17.42a | 3.69±0.20b | 355.08±24.35a | 356.85±34.61a | 0.9984 |
|  | S_CK | 251.33±12.08b | 3.74±0.20ab | 261.14±13.03b | 266.99±16.70b | 0.9994 |

Note: Different letters in the same column indicate significant differences at *p*<0.05 among the samples from same source.
